# Supplementary material for: Static Effective Hamiltonians for Molecular Systems through RPA-Based Downfolding
Source: J Chem Theory Comput. 2026 Sep 8;22(18):9468–82. doi: 10.1021/acs.jctc.6c01128 (PMC13602853; doi:10.1021/acs.jctc.6c01128)
Supplement: Supplementary file 1 [file ct6c01128_si_001.pdf]

Supporting Information for Static Effective Hamiltonians for Molecular Systems Through RPA-based Downfolding

Erik Verzijl and Arno Förster*

Department of Chemistry and Pharmaceutical Sciences, Vrije Universiteit, De Boelelaan 1105, 1081 HV Amsterdam, The Netherlands

E-mail: a.t.l.foerster@vu.nl

S1 N_2 with cc-pVTZ

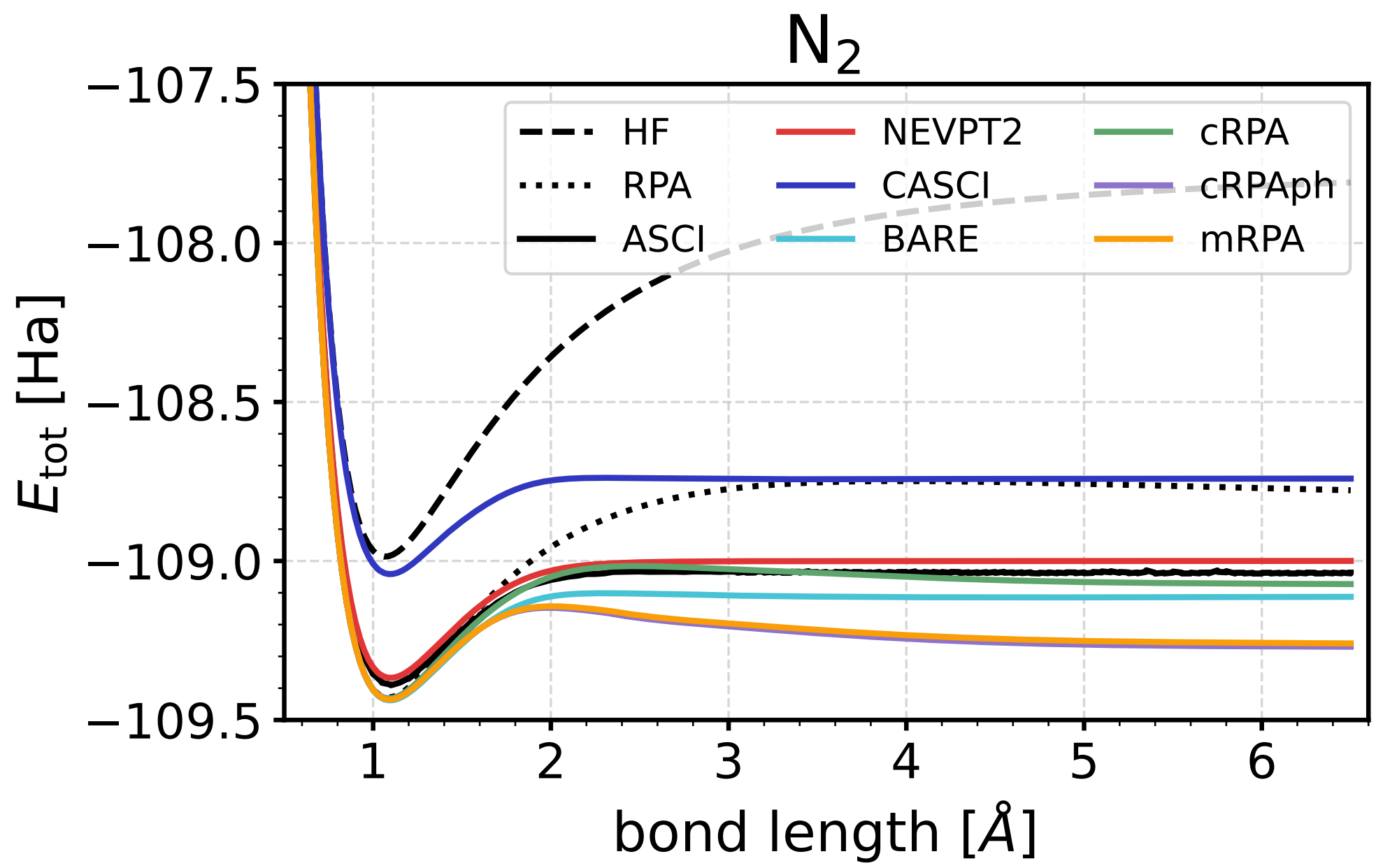

Fig. S1: N_2 in cc-pVTZ basis with a (6,6) active space, composed of all $2p$ linear combinations. The ASCI curve is obtained with $N_{\text{core}} = 32.000$ and $N_{\text{target}} = 320.000$.

Table S1: Equilibrium bond length in Åfor N_2 in cc-pVTZ and (6,6) active space.

ASCI	HF	RPA	CASCI	NEVPT2	BARE	cRPA	mRPA	cRPAph
1.100	1.070	1.080	1.100	1.100	1.095	1.089	1.093	1.092

Table S2: Bonding energy errors relative to FCI/ASCI reference in kcal/mol for N_2 in cc-pVTZ and (6,6) active space.

HF	RPA	CASCI	NEVPT2	BARE	cRPA	mRPA	cRPAph
517.8	188.4	−219.4	10.2	−16.4	5.0	−109.9	−116.8

Fig. S1 shows the dissociation curves for N_2 in cc-pVTZ basis. The ASCI curve shows some noise at larger bond lengths, which could be removed by enlarging the wavefunction, which would let the energy converge to FCI. If done for each point along the dissociation curve, the noise would vanish and the curve would be smoothed. However, because this

scales the compute time and does not change the qualitative picture, we kept the size of the wavefunction relatively small, as 320.000 determinants already leads to high compute times.

Table S1 and S2 show the interpolated equilibrium bond lengths and bonding energy error per method, respectively.

S2 Relative bonding energies

Fig. S2 shows energy curves relative to the energy at dissociation. The FCI/ASCI curves show the exact bonding energy within the basis set. HF is known to give nonphysically high bonding energies, which we also observe. RPA improves upon this, but still fails to be accurate. Generally, NEVPT2 shows the closest agreement with FCI/ASCI. CASCI generally gives too shallow wells for the equilibrium, which is accounted for if the RPA dynamical correlation is included, even with bare interactions. mRPA and cRPAph are shown to be inaccurate, which can be explained by the fact that for those methods, at dissociation, $E^{\mathcal{E}}_{\mathrm{corr}}$ is too low, shifting the curves up. cRPA performs relatively well, but does suffer from the local maximum at medium bond length.

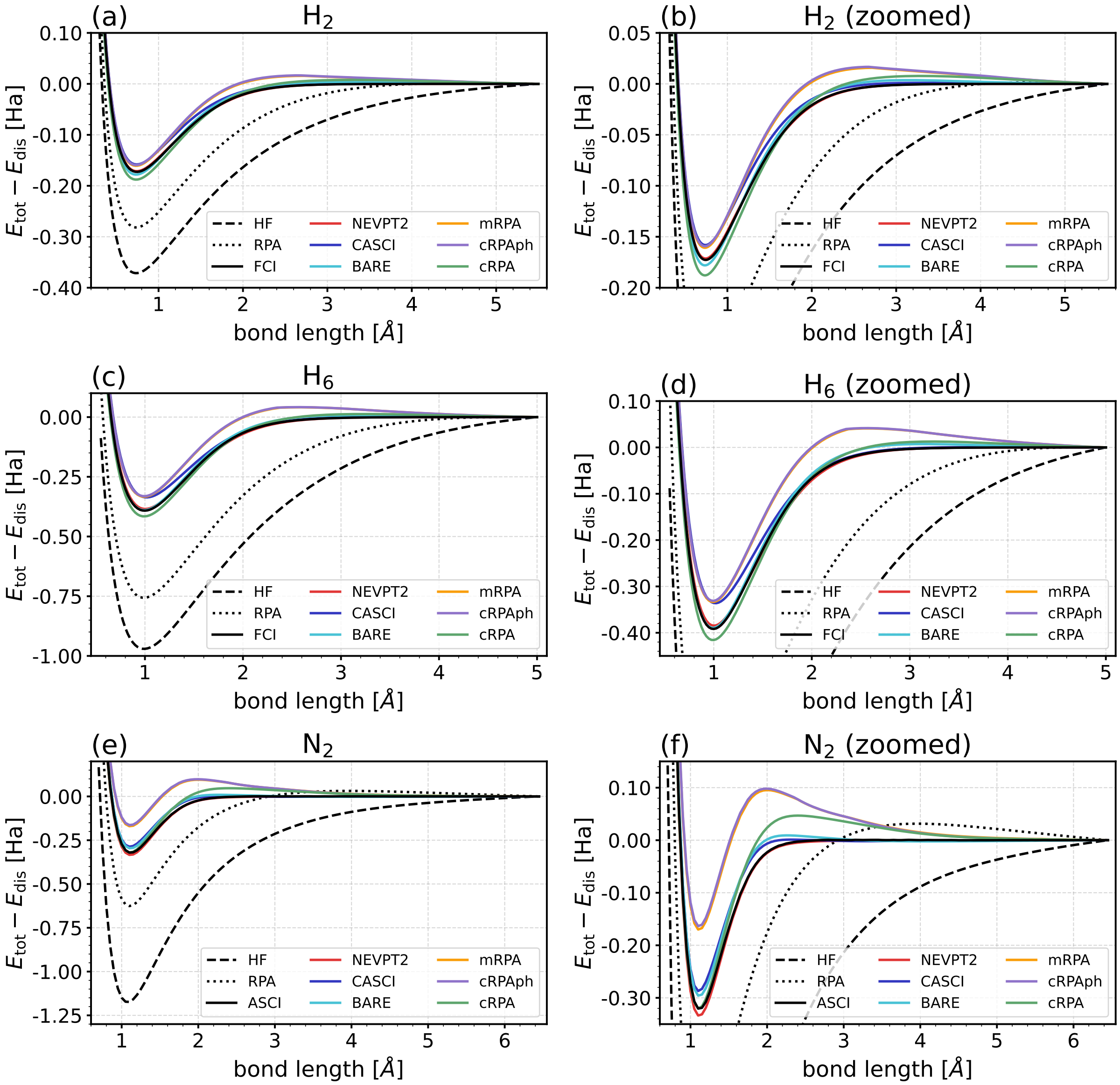

Fig. S2: Relative bonding energy curves of: a/b) H_2 (HF-orbitals) in cc-pVTZ basis, (2,2) active space. c/d) H_6 in cc-pVTZ basis, (6,6) active space. Bond length is the distance between neighboring hydrogen atoms, which is equal for all six pairs. e/f) N_2 in cc-pVDZ basis with a (6,6) active space, composed of all $2p$ linear combinations. The ASCI curve is obtained with $N_{\text{core}} = 32.000$ and $N_{\text{target}} = 320.000$.
