# Supplementary material for: Static Effective Hamiltonians for Molecular Systems through RPA-Based Downfolding
Source: J Chem Theory Comput. 2026 Sep 8;22(18):9468–82. doi: 10.1021/acs.jctc.6c01128 (PMC13602853; doi:10.1021/acs.jctc.6c01128)
Supplement: Supplementary file 2 [file ct6c01128_si_002.zip › Supplementary_Material/plots/SI2a_H2Bonding.pdf]

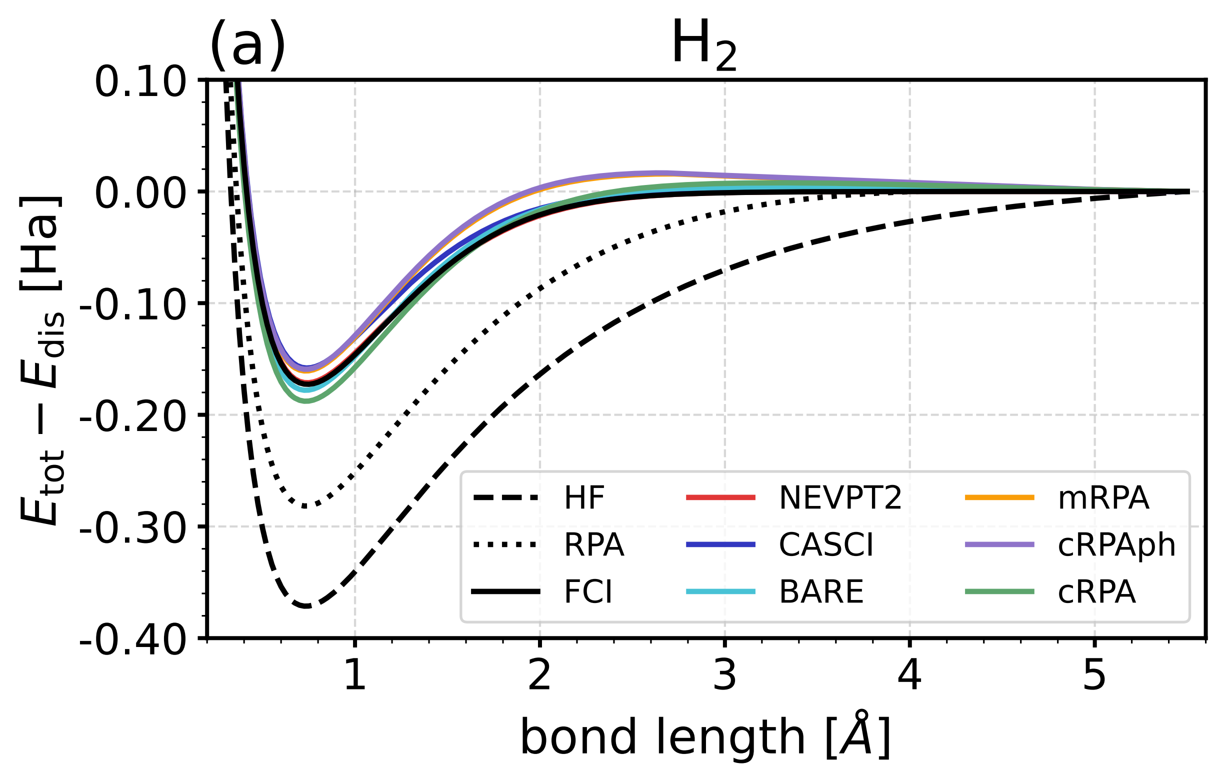

(a)
H_2
0.10
0.00
-0.10
-0.20
-0.30
-0.40
$E_{\mathrm{tot}} - E_{\mathrm{dis}}$ [Ha]
1
2
3
4
5
bond length [$\AA$]
HF
RPA
FCI
NEVPT2
CASCI
BARE
mRPA
cRPAph
cRPA
