# Supplementary material for: Static Effective Hamiltonians for Molecular Systems through RPA-Based Downfolding
Source: J Chem Theory Comput. 2026 Sep 8;22(18):9468–82. doi: 10.1021/acs.jctc.6c01128 (PMC13602853; doi:10.1021/acs.jctc.6c01128)
Supplement: Supplementary file 2 [file ct6c01128_si_002.zip › Supplementary_Material/plots/SI1_N2DissociationTZ.pdf]

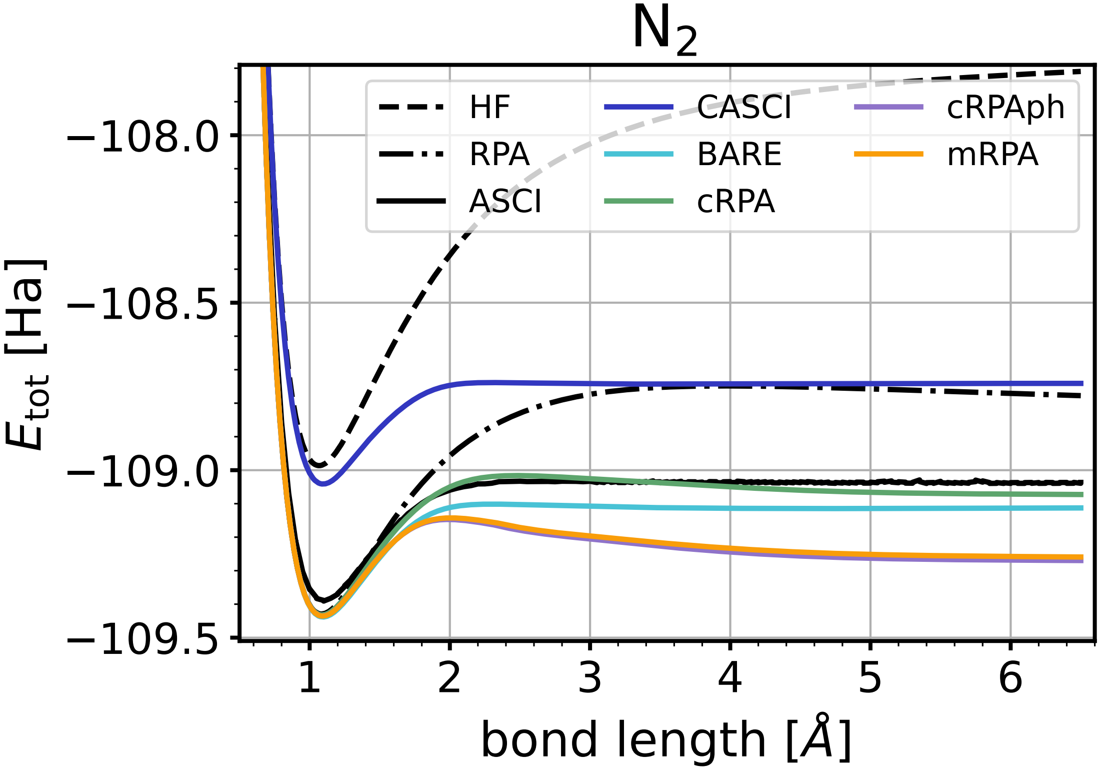
N2
HF
RPA
ASCI
CASCI
BARE
cRPA
cRPAph
mRPA
−108.0
−108.5
−109.0
−109.5
E_tot [Ha]
1
2
3
4
5
6
bond length [Å]
