# Supplementary material for: Static Effective Hamiltonians for Molecular Systems through RPA-Based Downfolding
Source: J Chem Theory Comput. 2026 Sep 8;22(18):9468–82. doi: 10.1021/acs.jctc.6c01128 (PMC13602853; doi:10.1021/acs.jctc.6c01128)
Supplement: Supplementary file 2 [file ct6c01128_si_002.zip › Supplementary_Material/plots/SI2e_N2Bonding.pdf]

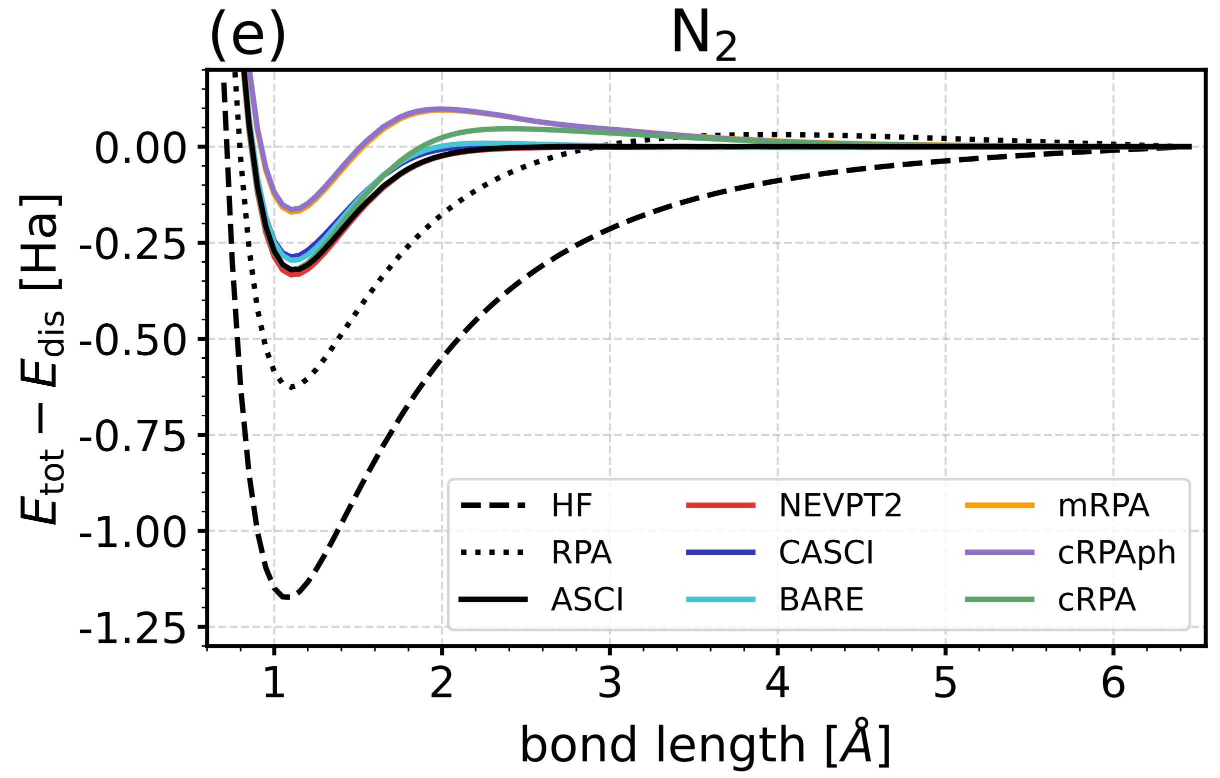

(e)
N_2
$E_{tot} - E_{dis}$ [Ha]
0.00
-0.25
-0.50
-0.75
-1.00
-1.25
1
2
3
4
5
6
bond length [Å]
HF
RPA
ASCI
NEVPT2
CASCI
BARE
mRPA
cRPAph
cRPA
