# Supplementary material for: Static Effective Hamiltonians for Molecular Systems through RPA-Based Downfolding
Source: J Chem Theory Comput. 2026 Sep 8;22(18):9468–82. doi: 10.1021/acs.jctc.6c01128 (PMC13602853; doi:10.1021/acs.jctc.6c01128)
Supplement: Supplementary file 2 [file ct6c01128_si_002.zip › Supplementary_Material/plots/SI2d_H6Bonding.pdf]

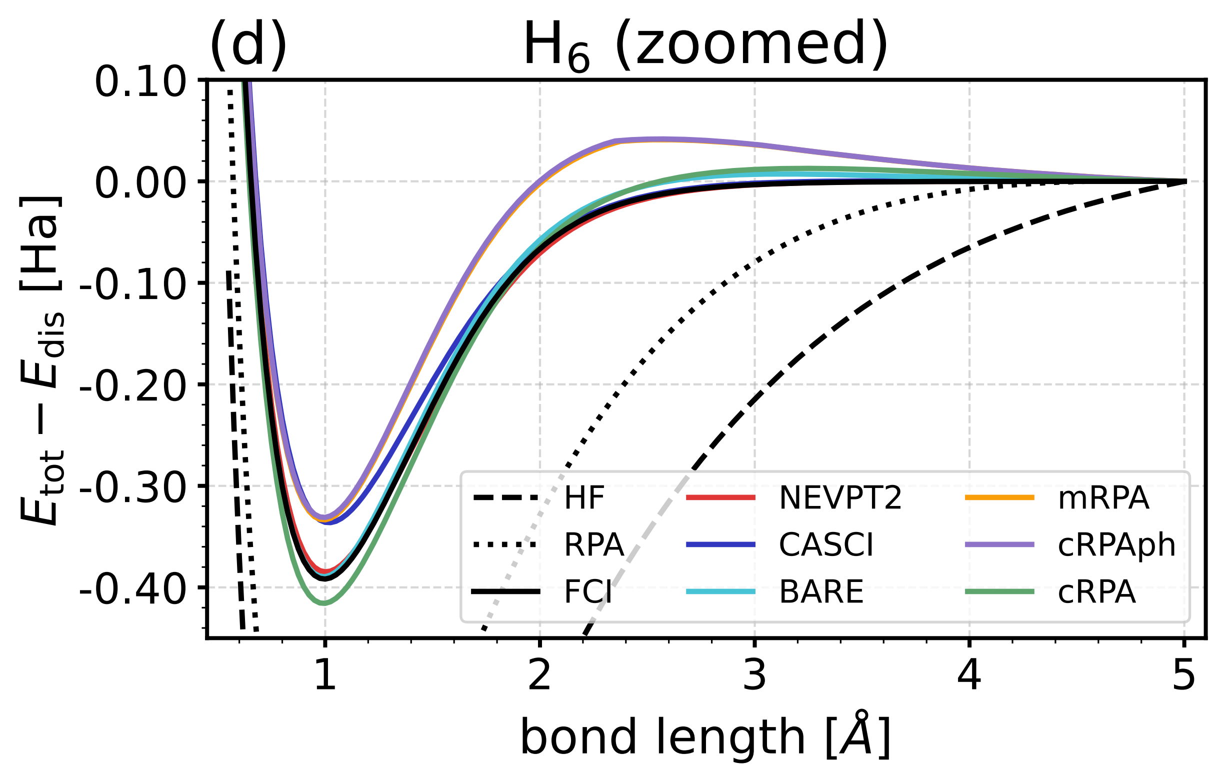

(d)
H6 (zoomed)
0.10
0.00
-0.10
-0.20
-0.30
-0.40
Etot − Edis [Ha]
1
2
3
4
5
bond length [Å]
HF
RPA
FCI
NEVPT2
CASCI
BARE
mRPA
cRPAph
cRPA
